# Arterial Hypotension Following Norepinephrine Decrease in Septic Shock Patients Is Not Related to Preload Dependence: A Prospective, Observational Cohort Study

**DOI:** 10.3389/fmed.2022.818386

**Published:** 2022-02-22

**Authors:** Stefan Andrei, Maxime Nguyen, Osama Abou-Arab, Belaid Bouhemad, Pierre-Grégoire Guinot

**Affiliations:** ^1^Anaesthesiology and Critical Care Department, Dijon Bourgogne University Hospital, Dijon, France; ^2^Anaesthesiology and Intensive Care Department, Carol Davila University of Medicine and Pharmacy, Bucharest, Romania; ^3^University of Burgundy Franche Comté, Dijon, France; ^4^Anaesthesiology and Critical Care Department, Amiens Picardie University Hospital, Dijon, France

**Keywords:** norepinephrine weaning, septic shock, volume therapy, preload responsiveness, dynamic arterial elastance

## Abstract

**Background:**

The optimal management of hypotensive patients during norepinephrine weaning is unclear. The primary study aim was to assess the ability of preload dependence to predict hypotension following norepinephrine weaning. The secondary aims were to describe the effect of norepinephrine weaning on preload dependence, and the cardiovascular effects of fluid expansion in hypotensive patients following norepinephrine weaning.

**Materials and Methods:**

This was a prospective observational monocentric study. We included PiCCO®-monitored patients with norepinephrine-treated septic shock, for whom the physician decided to decrease the norepinephrine dosage during the de-escalation phase. Three consecutive steps were evaluated with hemodynamic measurements: baseline, after norepinephrine decrease, and after 500 mL fluid expansion.

**Results:**

Forty-five patients were included. Preload dependence assessed by stroke volume changes following passive leg raising was not predictive of pressure response to norepinephrine weaning [AUC of 0.42 (95%CI: 0.25–0.59, *p* = 0.395)]. After fluid expansion, there was no difference in the prior preload dependence between pressure-responders and non-pressure-responders (14 vs. 13%, *p* = 1). The pressure response to norepinephrine decrease was not associated with pressure response after fluid expansion (40 vs. 23%, *p* = 0.211).

**Conclusion:**

Hypotension following norepinephrine decrease was not predicted by preload dependence, and there was no association between arterial hypotension after norepinephrine decrease and fluid response.

## Introduction

Fluid therapy and norepinephrine are the main hemodynamic treatments for septic shock (1, 2). Once the patient starts recovering, vascular tone progressively improves, and the hemodynamic de-escalation phase begins (1, 3). Norepinephrine weaning is an important part of the therapeutic process when treating patients with septic shock (2, 4). Because norepinephrine acts on both alpha- and beta-adrenergic receptors, it modulates several components of the cardiovascular equilibrium: venous return, cardiac preload, inotropy, and arterial load (5–10). Because of these effects, an intuitive but uncertain corollary would be that decreasing norepinephrine decreases venous return and cardiac preload, and thus cardiac output (9, 11). In this way, it would be expected that decreasing norepinephrine could promote arterial hypotension in relation to a decrease in preload, and that preload dependence prior to norepinephrine weaning may be associated with arterial hypotension. Since acute circulatory failure may be associated with altered preload and vasomotor tone, and norepinephrine may have “fluid-like effects,” physicians may arbitrarily infuse fluid to wean off norepinephrine in the belief that it may improve the weaning process. This physiological background is not well-documented, and it may lead to a positive fluid balance, which is associated with worsening outcomes in intensive care unit (ICU) (12).

The objective of the present study was to assess the association between prior preload dependence and the decrease in blood pressure following norepinephrine weaning. The secondary objectives were to describe the effect of norepinephrine weaning on preload dependence and the cardiovascular effects of fluid expansion in hypotensive patients following norepinephrine weaning.

## Materials and Methods

### Ethics

The study protocol was approved by the local Ethics Committee (*Comité de Protection des Personnes Nord-Ouest II* CHU–Place V. Pauchet, 80054 AMIENS Cedex 1, 2011-46). All patients or their next of kin provided informed consent to participation. This study was conducted over an 18-month period in the intensive care unit of the department of anesthesia and critical care of the University Hospital of Amiens, France.

### Patients

This is a prospective observational study. We included non-consecutive patients diagnosed with septic shock and treated with norepinephrine, for whom the attending physician decided to decrease the norepinephrine dosage, and who were monitored with a PiCCO® monitoring device. Sepsis was defined according to the International Sepsis Definitions Conference. Patients treated with epinephrine and/or dobutamine, patients with arrhythmia, or intra-abdominal hypertension, and individuals younger than 18 years were excluded. The present manuscript was drafted in compliance with the Strengthening the Reporting of Observational Studies in Epidemiology checklist for cohort studies.

### Hemodynamic Parameters Measurements

All patients were monitored with a central venous pressure (CVP) and femoral arterial catheter thermodilution system connected to a PiCCO® (PV2024, PULSION Medical Systems – GETINGE). Cardiac index (CI, L min^−1^ m^−2^), indexed global end diastolic volume (GEDI, mL m^−2^) and cardiac function index (CFI) were measured using transpulmonary thermodilution, with the injection of three 15-mL cold saline boluses. Systolic, mean and diastolic arterial pressure (SAP, MAP, and DAP) were measured from the arterial catheter. Total peripheral resistance (TPR) was calculated as MAP-CVP/CO (mmHg L^−1^ min^−1^). Arterial compliance was calculated as SV/arterial pulse pressure (PP) (mL mmHg^−1^) (13). Effective arterial elastance was calculated as Ea = MAP/SV (10, 14). Left ventricular elastance (E_V_) was approximated by the following formula: Ev = MAP/(GEDV/4-SV) (15). The dynamical arterial elastance was calculated as PP Variation/SV Variation (16).

### Study Design

Patients were evaluated at three consecutive steps. At baseline, the first hemodynamic measurements were performed with passive leg raise (PLR). The second measurements were performed after norepinephrine was decreased. Then, the third measurements were performed after fluid expansion. Thermodilution calibration was performed at each step. Decisions regarding the decrease in norepinephrine dosage and fluid expansion were left to the physician's discretion. Norepinephrine decrease was standardized for all patients at 0.04 μg kg^−1^ min^−1^. Only a one-step norepinephrine dose reduction was assessed in this study. Fluid expansion consisted of 500 mL of saline solution over a period of 10 min.

All patients had mechanical ventilation in volume-controlled mode and were sedated. Ventilator settings (inspired oxygen fraction, tidal volume, respiratory rate, and positive end-expiratory pressure) were not modified during the study period.

### Statistical Analyses

In absence of previous data, we performed an observational study with a convenience cohort of 45 patients. The distribution of variables was assessed using histograms, QQ plots, and the Shapiro-Wilk test. Data are expressed as numbers, proportions (in percent), medians [25–75% interquartile range], or as means (± standard deviation), as appropriate. Pressure non-responders and responders were defined by MAP variation (expressed as a percentage) after decreasing the dose of norepinephrine. A positive response was defined as a ≥10% decrease in MAP. Qualitative data were compared with a chi-squared test or Fisher's test, and quantitative data were assessed with a student's *t*-test or Mann-Whitney test, as appropriate. Paired data were compared with paired Student's *t*-test or Wilcoxon signed rank test. A receiver-operating characteristic (ROC) curve was constructed to assess SV changes following PLR, and dynamic arterial elastance to predict pressure response. The threshold for statistical significance was corrected by using Bonferroni adjustment for multiple comparisons and set to *p* < 0.025. Statistical analysis was performed by using RStudio (Version 1.1.447 – © 2009-2018 RStudio, Inc.).

## Results

**Figure 1:**
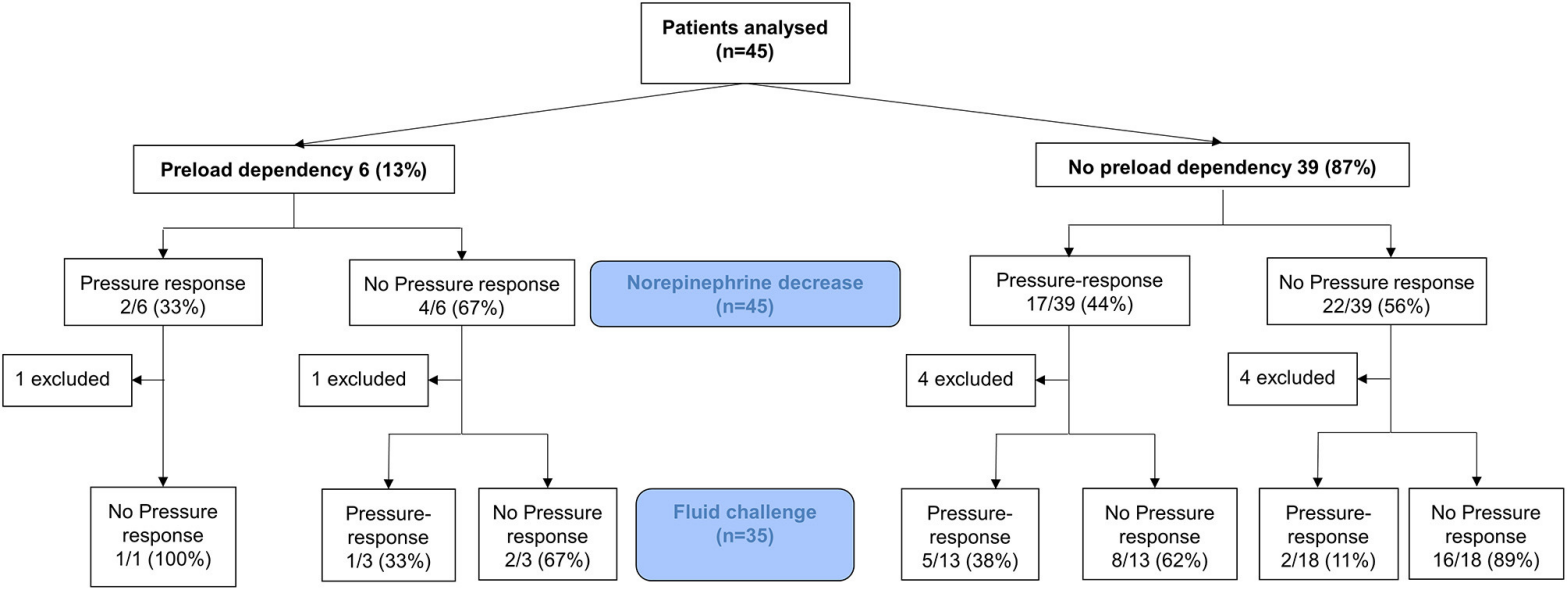
Flow chart of the study.

Of the 900 patients admitted to our ICU during the study period, forty-five patients were included and analyzed in the study (Figure 1). Their baseline characteristics are summarized in Table 1. Median age was 67 [60; 78] years, 36% were females, and the median SAPS II score was 55 [45; 63].

**Table 1:** Patient characteristics.

**Variables**	**All patients (*N* = 45)**
Age (year), median [IQR]	67 [60;78]
Men, *n* (%)	29 (64%)
BMI (Kg m^−2^), median [IQR]	27.8 [24.2;34.1]
SAPS II, median [IQR]	55 [45;63]
**Etiology of septic shock**, ***n*** **(%)**	
-Lung	21 (47%)
-Abdominal	18 (40%)
-Endocarditis	4 (9%)
-Blood stream infection	2 (4%)
Ventilator settings, median [IQR]	
-Tidal volume (mL kg^−1^)	8 [7;8]
-Plateau pressure (cmH_2_0)	22 [18.5–26]
-PEEP (cmH_2_0)	5 [5;10]
LVEF (%), median [IQR]	55 [50;60]
Norepinephrine (μg kg^−1^ min^−1^), median [IQR]	0.28 [0.12;0.60]

*IQR, interquartile range; BMI, body mass index; SAPS II, severe acute physiology score II; LVEF, left ventricle ejection fraction; ARDS, acute respiratory distress syndrome; PEEP, positive end-expiratory pressure*.

### Baseline and Response to Norepinephrine Decrease

The median decrease in norepinephrine dosage was 0.04 [0.03; 0.05] μg kg^−1^ min^−1^ for the entire cohort, and, respectively, 0.04 [0.02; 0.04] μg kg^−1^ min^−1^ for pressure responders, 0.04 [0.03; 0.05] μg kg^−1^ min^−1^ for pressure non-responders.

**Figure 2:**
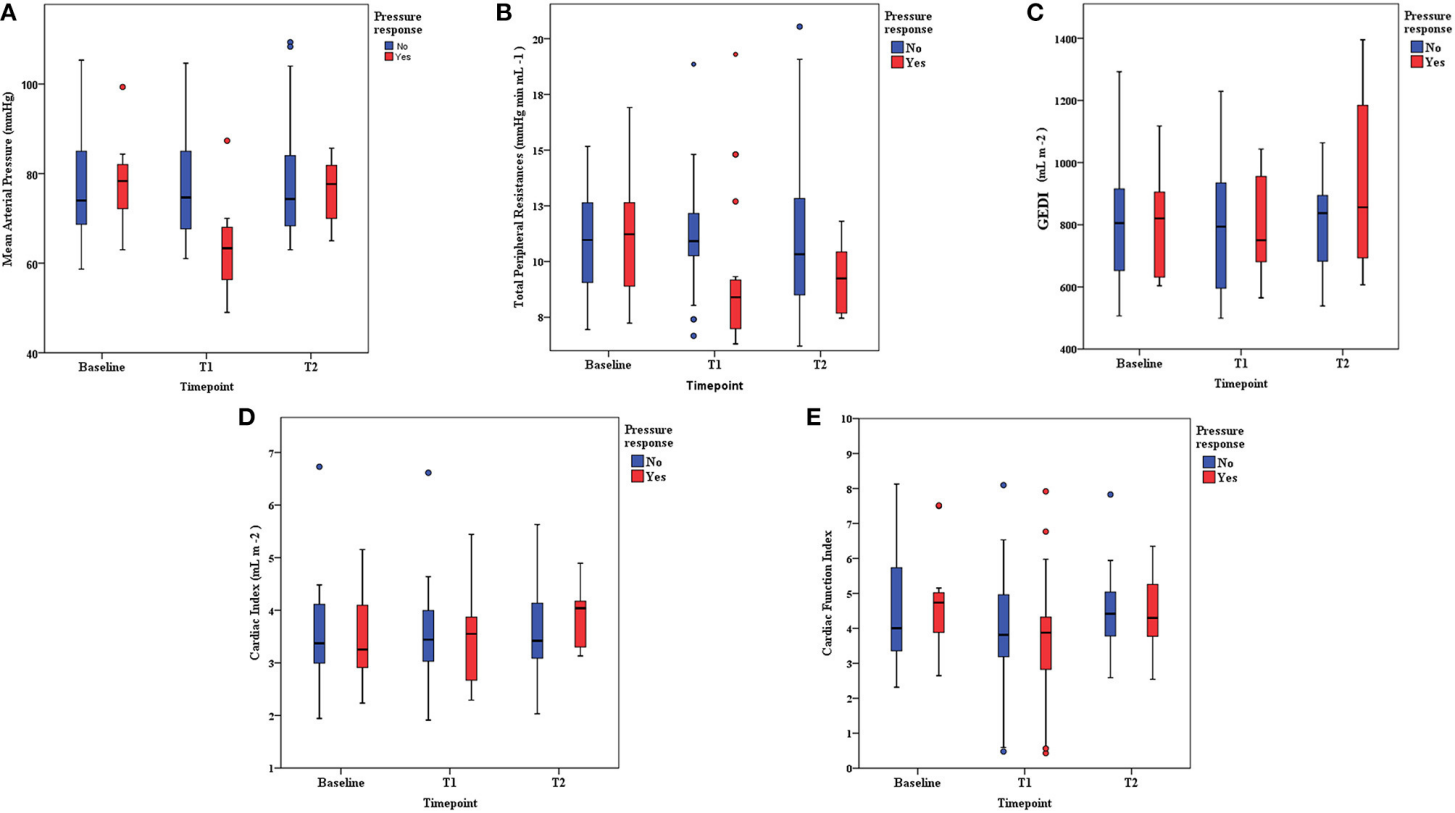
**(A)** Mean arterial pressure evolution from baseline to each intervention. Pressure responders vs. non-responders. T1 = norepinephrine dosage decrease. T2 = Fluid expansion. **(B)** TPR (total peripheral resistances) evolution from baseline to each intervention. Pressure responders vs. non-responders. T1 = norepinephrine dosage decrease. T2 = Fluid expansion. **(C)** GEDI (indexed global end diastolic volume) evolution from baseline to each intervention. Pressure responders vs. non-responders. T1 = norepinephrine dosage decrease. T2 = Fluid expansion. **(D)** Cardiac index evolution from baseline to each intervention. Pressure responders vs. non-responders. T1 = norepinephrine dosage decrease. T2 = Fluid expansion. **(E)** CFI (cardiac function index) evolution from baseline to each intervention. Pressure responders vs. non-responders. T1 = norepinephrine dosage decrease. T2 = Fluid expansion.

A decrease in the norepinephrine dose was associated with decreases in blood pressure (Figure 2A), and arterial tone (total peripheral resistances (Figure 2B), arterial elastance), whereas the other preload indices (CVP, PPV, SVV, GEDI) did not change (Table 2 and Figure 2C). The indicators of inotropy (CFI, Ev) decreased, but the CI did not (Figures 2D,E). Nineteen (42%) patients were classified as pressure responders. At baseline, 6 (13%) patients had a positive PLR with a median SV change of 10% [10–13]. Of the pressure responders, only 2 (11%) were PLR positive at baseline. There was no association between SV changes following PLR and pressure response to norepinephrine weaning (*p* = 1.0). After norepinephrine decrease, seven patients had a significantly decreased SV (by a mean of 2 ± 4%), and they were not associated with prior positive PLR (0% vs. 22%, *p* = 0.569) (Figure 1). With an AUC of 0.42 (95%CI 0.25–0.59, *p* = 0.395) SV during PLR did not predict pressure responders for norepinephrine weaning.

**Table 2:** Hemodynamic evolution following hemodynamic interventions.

**Variables**	**Baseline *N* = 45**	**NE decrease *N* = 45**	**Fluid expansion *N* = 35**
**HR (BPM**)			
Overall Pressure responder Pressure non-responder	87 [78;105] 79 [73;94.0] 92 [81;105]	85 [77;103] 78 [74;93] 91 [82;108]	85 [78;101] 82 [77;88] 89 [78;105]
**SAP (mmHg)**			
Overall Pressure responder Pressure non-responder	119 ± 12 121 ± 11 118 ± 13	109 ± 15^**$**^ 98 ± 12^**$**^ 115 ± 14	119 ± 15^*^ 118 ± 10^*^ 120 ± 17^*^
**DAP (mmHg)**			
Overall Pressure responder Pressure non-responder	53 [51;59] 56 [53;60] 52 [49; 67]	50 [45;57]^**$**^ 45 [40;50]^**$**^ 52 [47;68]	53 [49;63]^*^ 58 [48;61]^*^ 53 [49;65]^*^
**MAP (mmHg)**			
Overall Pressure responder Pressure non-responder	77 ± 10 78 ± 8 77 ± 12	71 ± 12^**$**^ 62 ± 9^**$**^ 77 ± 12	78 ± 12^*^ 76 ± 7^*^ 78 ± 13^*^
**CVP (mmHg)**			
Overall Pressure responder Pressure non-responder	11 ± 5 12 ± 5 10 ± 5	11 ± 5 11 ± 5^**$**^ 10 ± 4	13 ± 5^*^ 13 ± 6 13 ± 4^*^
**SV changes following PLR (%) or fluid challenge (%)**			
Overall Pressure responder Pressure non-responder	3 [1;8] 2 [0;8] 5 [1;8]	NA NA NA	9 [4;14] 10 [3;18] 9 [4;13]
**PPV (%)**			
Overall Pressure responder Pressure non-responder	10 [6;12] 7 [3;11] 10 [8;15]	10 [5;14] 9 [5;13] 12 [5;15]	8 [5;18] 7 [5;15] 10 [7;19]
**SVV (%)**			
Overall Pressure responder Pressure non-responder	10 [7;13] 8 [5;14] 10 [7;13]	12 [7;16] 10 [7;15] 12 [7;19]	9 [7;18] 10 [8;14] 9 [7;20]
**GEDI (mL m** ^**−2**^ ***)***			
Overall Pressure responder Pressure non-responder	806 ± 161 805 ± 172 806 ± 203	796 ± 174 789 ± 160 792 ± 197	837 ± 196^*^ 934 ± 301^*^ 807 ± 149^*^
**SV (mL)**			
Overall Pressure responder Pressure non-responder	69 ± 21 72 ± 22 66 ± 19	68 ± 21 71 ± 22 66 ±19	72 ± 21^*^ 85 ± 15^*^ 69 ± 21^*^
**Cardiac index (L min**^**−1**^ **m**^**−2**^**)**			
Overall Pressure responder Pressure non-responder	3.4 ± 0.7 3.5 ± 0.9 3.5 ± 0.9	3.3 ± 0.7 3.5 ± 0.9 3.5 ± 0.9	3.6 ± 0.8^*^ 3.9 ± 0.6^*^ 3.6 ± 0.9^*^
**Ea (mmHg mL** ^**−1**^ **)**			
Overall Pressure responder Pressure non-responder	1.13 [0.98;1.36] 1.04 [0.86;1.34] 1.22 [1;1.37]	1.12 [0.82;1.32]^**$**^ 0.89 [0.69;1.02]^**$**^ 1.16 [0.97;1.37]	1.06 [0.88;1.24] 0.93 [0.82;1.09] 1.1 [0.93;1.2]
**Dynamic arterial elastance (unit)**			
Overall Pressure responder Pressure non-responder	0.93 ± 0.3 0.75 ± 0.1 1.1 ± 0. 3	0.89 ± 0.2 0.84 ± 0.3^**$**^ 0.94 ± 0.2	0.86 ± 0.2 0.92 ± 0.4 0.83 ± 0.2
**TPR (mmHg min mL** ^**−1**^ **)**			
Overall Pressure responder Pressure non-responder	11 [9;13] 11 [9;13] 11 [9;12]	10 [8;12]^**$**^ 8 [7;9]^**$**^ 11 [10;12]	9 [8;12] 9 [8;10] 10 [8;13]
**Ca (mL mmHg** ^**−1**^ **)**			
Overall Pressure responder Pressure non-responder	1.12 ± 0.34 1.11 ± 0.33 1.12 ± 0.36	1.27 ± 0.43^**$**^ 1.37 ± 0.41^**$**^ 1.20 ± 0.44	0.77 ± 1.3 1.36 ± 0.31 0.79 ± 1.26
**Ev**			
Overall Pressure responder Pressure non-responder	0.40 [0.32;0.50] 0.41 [0.33;0.52] 0.36 [0.32;0.49]	0.36 [0.30;0.43]^**$**^ 0.34 [0.29;0.42]^**$**^ 0.36 [0.31;0.51]	0.26 [0.22;0.35] 0.26 [0.22;0.32] 0.26 [0.23;0.37]
**CFI**			
Overall Pressure responder Pressure non-responder	4.5 [3.6;5.3] 4.7 [3.9;5] 4 [3.4;5.7]	3.8 [3;4.9]^**$**^ 3.9 [3;4.3]^**$**^ 3.8 [3.2;4.9]	4.4 [3.8;5.3]^*^ 4.4 [3.7;5.5] 4.2 [3.8;5]
**NE infusion rate (μg kg** ^**−1**^ **min** ^**−1**^ **)**			
Overall Pressure responder Pressure non-responder	0.28 [0.12;0.60] 0.20 [0.08;0.46] 0.38 [0.19;0.86]	0.23 [0.83;0.61]^**$**^ 0.17 [0.45;0.43]^**$**^ 0.34 [0.14;0.81]^**$**^	– – –

*1. NE decrease and 2. Fluid challenge*.

*NE, norepinephrine, SAP, systolic arterial pressure, MAP:, mean arterial pressure, DAP, diastolic arterial pressure, CVP, central venous pressure, NA, not applicable, SV, stroke volume, PPV, pulse pressure variation, GEDI, indexed global end diastolic volume, TPR, total peripheral resistances, Ca, arterial compliance, Ea: arterial elastance, Ev, ventricular elastance, CFI, cardiac function index*.

$ *Comparisons with baseline values, p < 0.025*.

* *Comparisons with values after norepinephrine weaning, p < 0.025*.

### Response to Fluid Challenge After Norepinephrine Decrease

After norepinephrine decrease, 35 patients underwent fluid expansion (Figure 1). The patients received 7.9 ± 0.8 mL kg^−1^ IBW crystalloids, with a similar volume between pressure responders (7.6 ± 0.7 mL kg^−1^ IBW) and non-pressure responders (7.9 ± 0.8 mL kg^−1^ IBW) (*p* = 0.174).

There was no difference in the prevalence of prior preload dependence (i.e., SV changes following PLR) between pressure responders and non-pressure responders (14 vs. 13%, *p* = 1), nor with SV response among these patients (*p* = 1). Among the 16 SV responders (i.e., SV increase over 10%) with fluid expansion, only two patients (13%) were preload dependent at baseline.

The pressure response to norepinephrine decrease was not associated with pressure response after fluid expansion [14 (40%) vs. eight patients (23%), *p* = 0.211], nor with SV response to fluid expansion (16 patients, 48%, *p* = 0.782). With an AUC of 0.81 (95%CI 0.63–0.97, *p* = 0.014), only dynamic arterial elastance predicted pressure response to fluid expansion.

## Discussion

This study demonstrated that prior preload dependence is not associated with arterial hypotension following norepinephrine weaning. Norepinephrine decrease was associated with cardiovascular effects on both cardiac inotropy and arterial load. Fluid expansion in arterial hypotensive patients following norepinephrine weaning did not systematically restore blood pressure despite increasing SV.

Few studies have focused on the hemodynamic effects and management of norepinephrine dosage decrease during the weaning process, after the resolution of the acute shock phase (17, 18). However, norepinephrine weaning in septic shock is not simply the reverse of the acute phase management. In the acute phase, it has been shown that the cardiovascular equilibrium depends on the underlying disease (i.e., sepsis vs. non sepsis), the type and dose of medications (inotrope, vasopressor, or both), and the fluid therapy provided during the resuscitation phase (1, 11, 13, 19–21). Several studies have demonstrated that norepinephrine can act as a fluid challenge by increasing venous return and cardiac pre-load (8, 9, 11). It would therefore seem intuitive that norepinephrine weaning mainly causes arterial hypotension by decreasing venous return, and that preload dependence prior to norepinephrine decrease should be associated with arterial hypotension. In the same way, it would be assumed that further fluid expansion should restore blood pressure. Our observations contradict these beliefs. Preload dependence prior to norepinephrine decrease was not associated with a higher incidence of hypotension following norepinephrine decrease. Moreover, fluid expansion did not significantly restore blood pressure after arterial hypotension due to norepinephrine decrease whereas the CI increased. Several points can be discussed in view of these observations, which demonstrate a non-preload phenomenon of arterial hypotension following norepinephrine decrease.

The decrease in blood pressure may be explained by the effects of norepinephrine on cardiac function and arterial load (6, 12, 19, 20). We did observe a decrease in CFI and ventricular elastance, which are two parameters of cardiac systolic function. Studies have demonstrated that the administration of a low dose of norepinephrine can increase left ventricular function, CI, and blood pressure (19, 21). These alterations may partially explain the decrease in blood flow and thus in blood pressure.

Blood pressure and blood flow depend preload effects of norepinephrine may depend on norepinephrine dose and acute shock phase. Most studies evaluating the preload effects of norepinephrine were performed in the initial phase of acute circulatory failure (9, 10). During this phase, it is likely that the ratio of preload dependence is higher than in later phases of resuscitation (21). Because patients may be more preload dependent during the resuscitation phase, norepinephrine may affect venous return and preload, and thus CI, more effectively. On the contrary, during the weaning phase, patients have been resuscitated and may be less preload dependent, so the effect of norepinephrine on preload may be lower. In this way, we observed a low prevalence of positive PLR tests, and low amplitude of change in cardiac preload parameters, with no significant change in CI.

In addition, the effects of norepinephrine on venous return and cardiac preload may be related to high doses and/or dose adjustments of norepinephrine. The mean norepinephrine dose adjustments required to demonstrate such effects was found to be higher in studies on norepinephrine weaning (7–9, 11). In our study, the dynamic/static preload indices did not change with small decreases in norepinephrine. We only observed significant changes in arterial load and inotropy. These effects have already been demonstrated in several studies. These authors have demonstrated the effects of norepinephrine on venous return and CI by using higher dose of norepinephrine than in the present study (7–9, 11). In view of the literature and our findings, we can hypothesize that the effects of norepinephrine on venous return and cardiac preload may related to higher doses and/or dose adjustments of norepinephrine. Norepinephrine has a fluid challenge effects but it may depend on the norepinephrine dose: at low dose main effect is expressed on arterial load and inotropy whereas at higher dose this effect is expressed also on the venous return. Overall, these observations are in line with the results of the SNEAD study, which demonstrated that despite a shorter norepinephrine support time, the intervention group did not receive more fluid than the control group (17). In this study, norepinephrine was decreased by small increments until total withdrawal (17).

### Clinical Implication and Future Perspectives

Vasopressor weaning is a dynamic process that requires a careful evaluation of the different components of the cardiovascular system (3). Physicians should consider the clinical benefit of fluid expansion in arterial hypotension during norepinephrine weaning. Fluid expansion should not be considered prior to weaning, even in preload dependent patients. Moreover, when arterial hypotension occurs after norepinephrine is decreased, fluid expansion will not systematically restore blood pressure. In order to better manage this clinical paradigm, dynamic arterial elastance was already proposed as a useful indicator that can indicate which patients will experience a blood pressure increase with a rise in CO (17, 22). In other words, fluid expansion should be considered only in preload patients with a high dynamic arterial elastance value (14, 23). If this is not the case, the physician should increase norepinephrine to the previously used dose. However, further bigger studies are needed to provide a fully clinical validation of this approach.

### Limitations

Firstly, the limitations of our study include its observational, non-randomized, monocentric, open design, and limited sample size. We performed an observational study without power calculation that included patients for whom the physician decided to wean off norepinephrine. Also, the volume of fluid expansion was fixed at 500 mL crystalloids, which is different from some other approaches (5 mL kg^−1^ of IBW). Even though this approach is closer to the daily life practice, a degree of subjectivity cannot be fully eliminated. Nevertheless, our results are in line with a randomized study that evaluated active norepinephrine weaning vs. standard norepinephrine weaning. Furthermore, the design of the study did not allow us to infer causality, and so association are provided in the present manuscript. Finally, it has to be pointed that the patients with septic shock (originating from different origins) are intrinsically complex, as the patients have various comorbidities, and many interventions are possibly performed, e.g. different amounts of fluid administration (crystalloids), albumin supplementation, renal replacement therapy, diuretic therapy. We assessed the E_V_ by using a simplified formula based on the assumption that atria and ventricle volume are equal (15). Such simplified formula may be imperfect and far away from gold standard measure of E_V_ (24, 25). Nevertheless, we observed the same changes of Ev and CFI that are two indirect measures of ventricular inotropy. All these potential confounders were considered in our study analyses.

## Conclusion

Arterial hypotension following norepinephrine decrease was not associated with preload dependence. Moreover, we found no association between arterial hypotension after norepinephrine decrease and fluid expansion response. Therefore, patients with arterial hypotension following norepinephrine decrease should not be systematically treated with fluid therapy.

## Data Availability Statement

The raw data supporting the conclusions of this article will be made available by the authors, without undue reservation.

## Ethics Statement

The studies involving human participants were reviewed and approved by Comité de Protection des Personnes Nord-Ouest II CHU - Place V. Pauchet, 80054 AMIENS Cedex 1, 2011-46. The patients/participants provided their written informed consent to participate in this study.

## Author Contributions

SA and P-GG are guarantors of the entire manuscript. OA-A, SA, MN, BB, and P-GG designed the study. OA-A and P-GG collected and analyzed all the data. All the authors helped in the data interpretation and the manuscript draft. All authors read and approved the final manuscript.

## Conflict of Interest

The authors declare that the research was conducted in the absence of any commercial or financial relationships that could be construed as a potential conflict of interest.

## Publisher's Note

All claims expressed in this article are solely those of the authors and do not necessarily represent those of their affiliated organizations, or those of the publisher, the editors and the reviewers. Any product that may be evaluated in this article, or claim that may be made by its manufacturer, is not guaranteed or endorsed by the publisher.
